# Supplementary material for: Novel Driver Strength Index highlights important cancer genes in TCGA PanCanAtlas patients
Source: PeerJ. 2022 Aug 11;10:e13860. doi: 10.7717/peerj.13860 (PMC9375969; doi:10.7717/peerj.13860)
Supplement: Supplemental Information 2 [file peerj-10-13860-s002.zip › COHORTS/genes plots/detailed/2021_8_16_14_9_distribution_events_detailed_males_38.pdf]

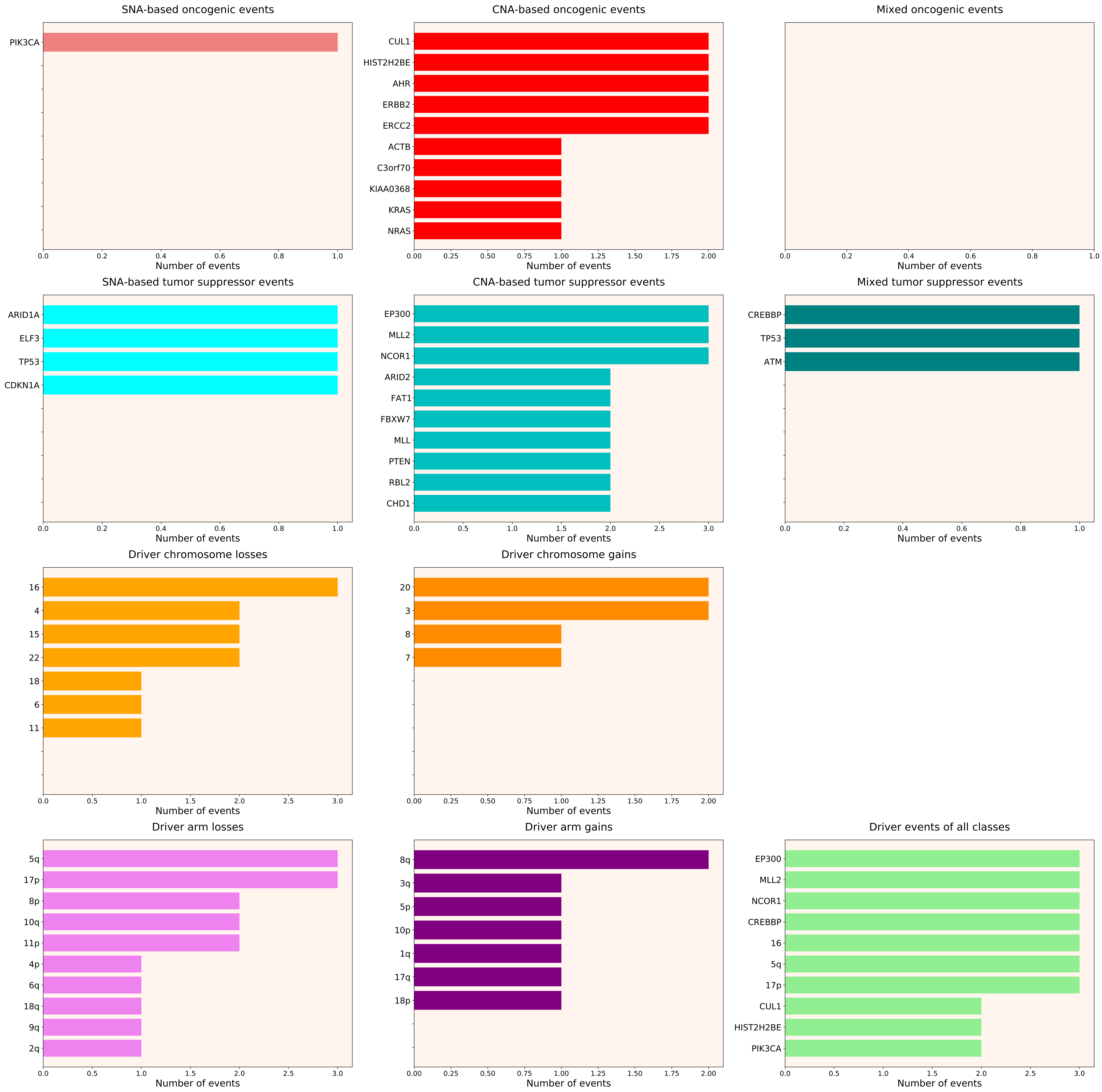
SNA-based oncogenic events
PIK3CA
Number of events
CNA-based oncogenic events
CUL1
HIST2H2BE
AHR
ERBB2
ERCC2
ACTB
C3orf70
KIAA0368
KRAS
NRAS
Number of events
Mixed oncogenic events
Number of events
SNA-based tumor suppressor events
ARID1A
ELF3
TP53
CDKN1A
Number of events
CNA-based tumor suppressor events
EP300
MLL2
NCOR1
ARID2
FAT1
FBXW7
MLL
PTEN
RBL2
CHD1
Number of events
Mixed tumor suppressor events
CREBBP
TP53
ATM
Number of events
Driver chromosome losses
16
4
15
22
18
6
11
Number of events
Driver chromosome gains
20
3
8
7
Number of events
Driver arm losses
5q
17p
8p
10q
11p
4p
6q
18q
9q
2q
Number of events
Driver arm gains
8q
3q
5p
10p
1q
17q
18p
Number of events
Driver events of all classes
EP300
MLL2
NCOR1
CREBBP
16
5q
17p
CUL1
HIST2H2BE
PIK3CA
Number of events
